# High Performance Li_4_Ti_5_O_12_/Si Composite Anodes for Li-Ion Batteries

**DOI:** 10.3390/nano5031469

**Published:** 2015-08-28

**Authors:** Chunhui Chen, Richa Agrawal, Chunlei Wang

**Affiliations:** Department of Mechanical and Materials Engineering, Florida International University, Miami, FL 33174, USA; E-Mails: cchen012@fiu.edu (C.C.); ragra005@fiu.edu (R.A.)

**Keywords:** lithium titanate, silicon, rate capability, energy capacity, composite

## Abstract

Improving the energy capacity of spinel Li_4_Ti_5_O_12_ (LTO) is very important to utilize it as a high-performance Li-ion battery (LIB) electrode. In this work, LTO/Si composites with different weight ratios were prepared and tested as anodes. The anodic and cathodic peaks from both LTO and silicon were apparent in the composites, indicating that each component was active upon Li^+^ insertion and extraction. The composites with higher Si contents (LTO:Si = 35:35) exhibited superior specific capacity (1004 mAh·g^−1^) at lower current densities (0.22 A·g^−1^) but the capacity deteriorated at higher current densities. On the other hand, the electrodes with moderate Si contents (LTO:Si = 50:20) were able to deliver stable capacity (100 mAh·g^−1^) with good cycling performance, even at a very high current density of 7 A·g^−1^. The improvement in specific capacity and rate performance was a direct result of the synergy between LTO and Si; the former can alleviate the stresses from volumetric changes in Si upon cycling, while Si can add to the capacity of the composite. Therefore, it has been demonstrated that the addition of Si and concentration optimization is an easy yet an effective way to produce high performance LTO-based electrodes for lithium-ion batteries.

## 1. Introduction

In recent years, the need for high performance lithium-ion batteries has increased dramatically given their potential application in electric vehicles (EVs) and hybrid electric vehicles (HEVs) [1,2,3,4,5]. Spinel-Li_4_Ti_5_O_12_ (LTO) has attracted significant interest as an anode material for lithium-ion batteries because of several advantages: as a zero-strain insertion material, LTO is able to intercalate three lithium ions per molecule with negligible volumetric changes (spinel Li_4_Ti_5_O_12_ to rock salt Li_7_Ti_5_O_12_) [6,7,8,9]. In addition, LTO exhibits a very flat charge-discharge at ~1.55 V *vs.* Li/Li^+^, which is well above the formation potential of solid electrolyte interface (SEI) [6,7,8,9]. The ability of LTO to intercalate lithium ions with almost negligible strain gives it excellent cycle longevity and the SEI-free operational potential makes it a safe material. However, low intrinsic electrical conductivity (*ca*. 10^−13^ S·cm^−1^) and poor lithium-ion diffusivity (*ca*. 10^−9^–10^−13^ cm^2^·s^−1^) are two major issues that need to be addressed in order to realize excellent performance from LTO-based electrodes [10]. Several strategies have been adopted to address these challenges. For instance, conductive surface coatings [11,12,13] and cation doping [14,15] have been used to enhance the electrical conductivity of LTO while “nano”-sizing of LTO has shown to improve the specific capacity due to the reduction in both electron and ion-transport distances [16,17,18,19,20,21].

The theoretical capacity of structural transition of LTO from spinel to rock salt is only 175 mAh·g^−1^, if the cell is discharged to 1 V [11]. However, it has been shown that discharging LTO to 0 V allows for further intercalation of additional 1.5 lithium ions and as a result of which the theoretical capacity reaches 298 mAh·g^−1^ [22,23,24]. This not only enhances the overall energy density because of the larger theoretical capacity but also because of the lowered working voltage. However, discharging LTO to 0 V induces the possibility of SEI formation which in general is detrimental to the electrode performance. An intuitive approach to increase the capacity of LTO is making composites with anode materials with superior theoretical capacities. However, for most anode materials the operational voltages are less than 1 V, which makes discharging the composite below 1 V a prerequisite. In some of the reports, Sn [25], SnO_2_ [26], CuO [27] and Fe_2_O_3_ [28] were used with LTO and improvement in specific capacity was noted. Since the enhancement in capacity is a direct result of the composite components, silicon makes an excellent choice given its phenomenal theoretical capacity (4200 mAh·g^−1^), which is about 13 times higher than that of LTO (even when discharged to 0 V) [29]. However, one of the major drawbacks of silicon is the ~300% volumetric change upon charging and discharging, which results in peeling and pulverization of the electrode and eventual capacity loss. Numerous works have been carried out to address these issues, such as using nano-enabled silicon structures, preparing Si/C composites to improve electric conductivity and constructing porous structure or compositing with other active/non-active matrix to buffer the volume change [30,31,32,33]. Considering the structure stability of LTO, it is expected that LTO can also alleviate some of the volumetric expansion of silicon during the cycling process by acting as a buffer. Until now, only a few studies have been reported on the use of LTO/Si composite electrodes. Lin *et al.* [34] deposited a thin coating of amorphous silicon on the surface of LTO electrode by thermal evaporation aiming to improve the cycling performance of LTO at elevated temperatures. However, there is a lack of systematic study on LTO/Si composites for LIB electrodes.

In this paper, the goal is to investigate if LTO/Si composites hold potential as high-performance Li-ion battery anodes. We have evaluated the electrochemical performance of LTO/Si composites based on commercial battery grade electrode materials using conventional battery fabrication procedures. The synergism between LTO and Si was investigated by studying the influence of different weight ratios of LTO and Si on the rate capability and cyclability of the composites. Two control samples (pure-LTO, pure-Si) and three composite samples (65LTO5Si, 50LTO20Si, 35LTO35Si, the number indicates the weight percent of each component in the composite) were prepared and tested. It was found that sample 35LTO35Si delivered a capacity of 1004 mAh·g^−1^ at a current density of 0.22 A·g^−1^. Moreover, a capacity of 265 mAh·g^−1^ was maintained at a high current density of 2.25 A·g^−1^. Even at a very high current density of 7 A·g^−1^, sample 50LTO20Si were still able to deliver a stable capacity of 100 mAh·g^−1^ with good cycling performance. Our results show that the LTO/Si composites exhibit both improved energy capacity and high rate capability.

## 2. Results and Discussion

The LTO and Si composites were prepared with five different weight ratios, as summarized in Table 1. In this work, the current densities at different C-rates were calculated based on their theoretical values as well as the weight ratios of LTO and Si components. Poly (acrylic acid) (PAA) and super P Li^®^ were used as binder and conducting additive, respectively.

**Table 1:** Summary of LTO/Si composites.

Sample	Composition (wt %)				1 C Current Density ^a^ (mA·g^−1^)
	Si	LTO	PAA	Super P Li^®^	
pure-LTO	0	70	15	15	298
65LTO5Si	5	65	15	15	577
50LTO20Si	20	50	15	15	1413
35LTO35Si	35	35	15	15	2249
pure-Si	70	0	15	15	4200

Note: ^a^ Calculation based on theoretical capacity of LTO (298 mAh·g^−1^) and Si (4200 mAh·g^−1^).

**Figure 1:**
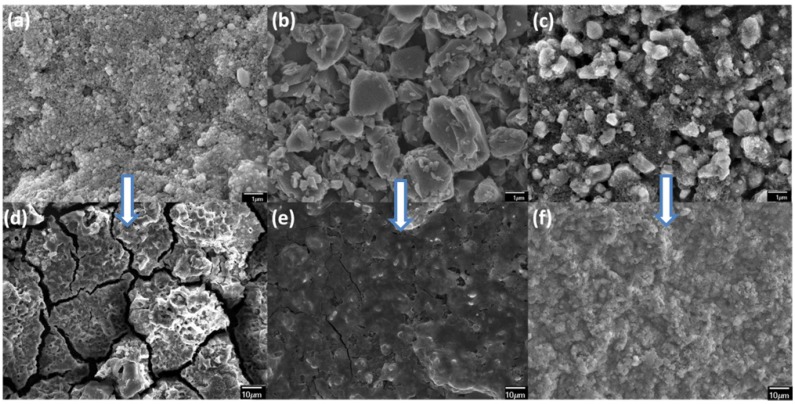
Typical SEM images of electrodes based on (**a**) pure-Si; (**b**) pure-LTO and 50LTO20Si before the electrochemical test (**a**–**c**) and after the electrochemical test (**d**–**f**), respectively.

The morphologies of typical as-casted LTO/Si composite electrodes are shown in Figure 1a–c. For pure Si in Figure 1a, it can be seen that the average size of silicon particles was around 100 nm and the particles were relatively uniformly mixed with other additives, which includes carbon black and polymer binder. From the pure LTO in Figure 1b, it can be observed that the LTO particles were relatively large with particle size ranging from 0.2 to 1 μm. The large size and irregular shape of LTO particles results in relatively low-density composites. Figure 1c shows the morphology of 50LTO20Si composite electrode. No obvious cracks were observed in the as-deposited electrodes. However, the particle size of LTO in 50LTO20Si is smaller than that in pure-LTO electrode, which may be due to the grounding effect of Si nanoparticles in the sample preparation process. Morphologies of the respective electrodes after electrochemical tests are shown in Figure 1d–f and will be discussed later.

**Figure 2:**
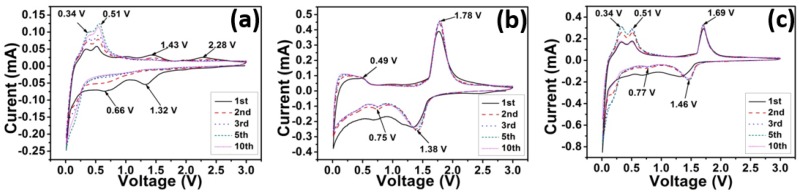
CV curves of electrodes based on (**a**) pure-Si, (**b**) pure-LTO, and (**c**) 50LTO20Si at scan rate of 0.2 mV s^−1^ in the range of 0.02–3 V, respectively.

Figure 2a exhibits the CV curves for the pure-Si electrode. A broad peak can be seen at around 0.4–0.95 V in the cathodic branch of first and second cycles. However, this broad peak disappears in the third cycle. No obvious oxidation peaks corresponding to this reduction process is observed in the anodic branch, which indicates that this peak corresponds to the solid electrolyte interphase (SEI) formation [32,33]. There is a distinctive reduction peak which starts at 0.3 V and becomes quite sharp below 0.1 V. This peak is attributed to the lithiation of silicon [32,33]. During charging, two well defined peaks centered at 0.34 and 0.51 V are observed and are ascribed to the formation of intermediate Li*_x_*Si and amorphous silicon, respectively [30,31]. The magnitude of current at these two anodic peaks increases from 0.05 to 0.12 mA from the first to the fifth cycle due to activation process from Si phase [35,36]. However, the peak current stays almost the same at the 10th cycle, implying that the activation process of Si is almost completed at the end of 10th cycle. The observed CV curves and the activation process are in good agreement with previous report of porous silicon [32,33,34,35,36]. The reconstruction of crystal structure near silicon surface is explained as the reason for the activation phenomenon [35,36].

Figure 2b displays the CV curves of pure-LTO electrode: a pair of redox peaks appears at 1.38 V (reduction) and 1.78 V (oxidation), which corresponds to the Li^+^ insertion and extraction process of LTO, which is in good agreement with literature [9,37]. The 0.5 V separation between the anodic and cathodic peaks is most likely because of the polarization resulting from the sluggish lithium diffusion and relatively low electrical conductivity [38,39]. Further Li intercalation into Li_7_Ti_5_O_12_ leads to a broad shoulder from 0.02 to 0.5 V. A small peak at around 0.75 V can be seen from the first to the 10th cycle, which is also due to the further lithium intercalation into the Li_7_Ti_5_O_12_ structure [9,37]. In the second cathodic cycle the current value dips as compared to the first cycle. This decrease in current is attributed to the SEI formation owing to the low operational voltage [38].

Figure 2c displays the CV curves for the 50LTO20Si composite electrode. As compared to Figure 2a,b, this electrode has both anodic and cathodic peaks for both Si and LTO as expected and the peak positions remain unchanged, which proves that both Si and LTO can work well with each other without interfering with the activity of the other component.

**Figure 3:**
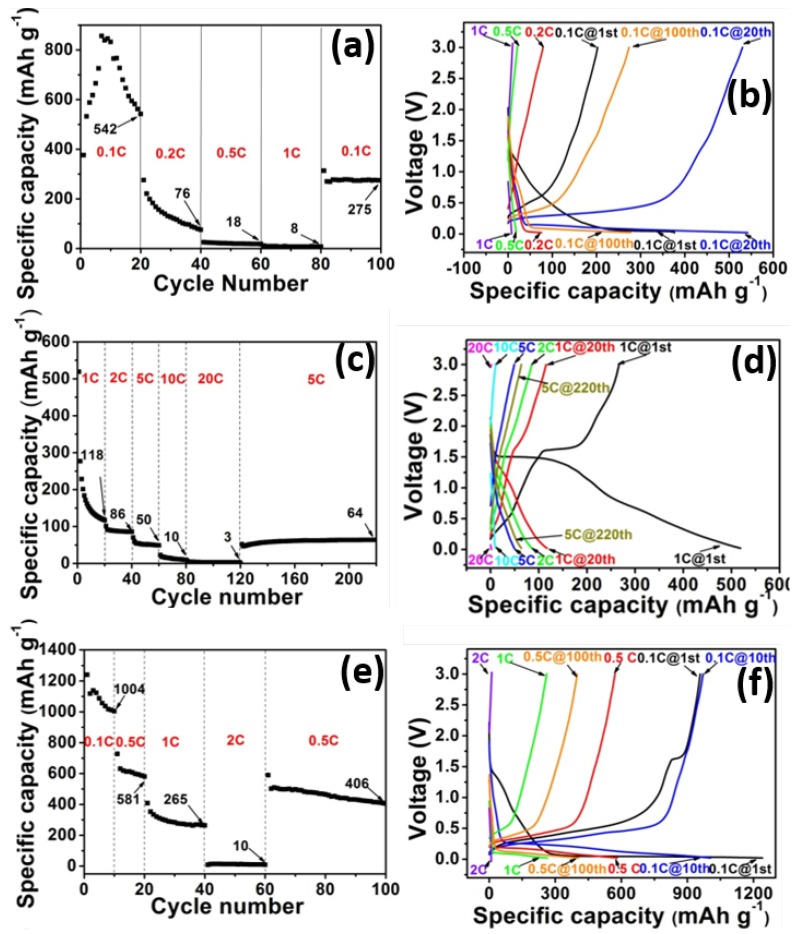

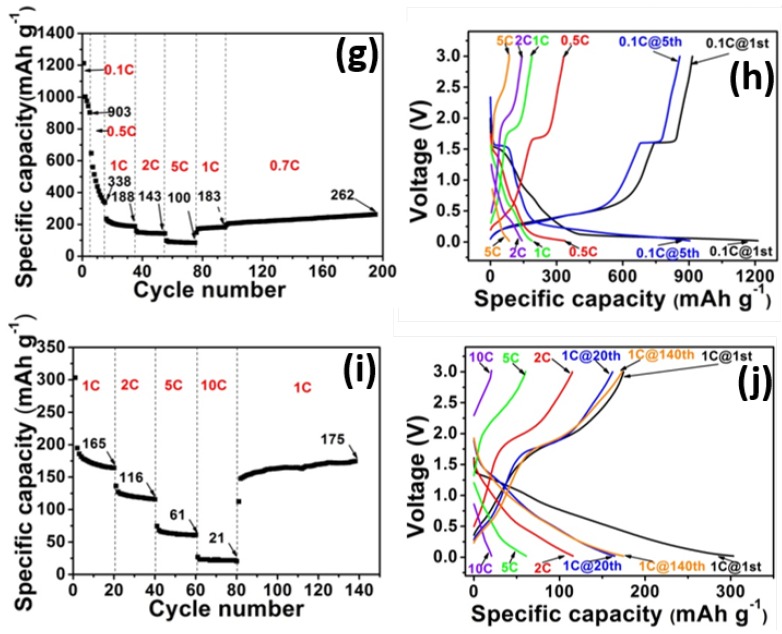
The rate performance and corresponding charge-discharge curves for electrodes based on pure-Si (**a**,**b**), pure-LTO (**c**,**d**), 35LTO35Si (**e**,**f**), 50LTO20Si (**g**,**h**) and 65LTO5Si (**i**,**j**), respectively.

In order to further examine the synergy between LTO and silicon, rate capability and cycling behavior, the composites were evaluated for different LTO:Si ratios. Figure 3 shows the rate performance of five different samples with different LTO:Si ratios and their corresponding charge/discharge curves. It can be seen from Figure 3a that the specific capacities of pure-Si sample at 0.1, 0.2, 0.5 and 1 C are 542, 76, 18 and 8 mAh·g^−1^, respectively. The first cycle discharge capacity is only 377 mAh·g^−1^, which gradually increases to a maximum value of 850 mAh·g^−1^ at the 7th cycle. The low discharge capacity during the first cycle is probably due to partial utilization of the silicon particles and the enhanced capacity in later cycles can be explained by the activation process in silicon [35,36], which is consistent with what has been observed in the CV curve. However, the capacity decreases to 542 mAh·g^−1^ at the 20th cycle, and most of the capacity is delivered between 0.05 and 0.2 V which can be seen in the corresponding charge-discharge curves. The capacity decreases as the C-rate increases and at 1 C, only 8 mAh·g^−1^ was achieved. When charged back to 0.1 C, a capacity of 275 mAh·g^−1^ was recovered, which stabilized at 100th cycle. In order to understand the capacity decrease and partial recovery of pure-Si electrode, the coin cell was disassembled after the rate capability tests at 100th cycle and structural changes in the electrode were examined, as shown in Figure 1d. Severe cracks were generated, which led to the isolation of some islands and failure of the electrode. In some areas, peeling was also observed (not shown in Figure 1), which is a common problem for pristine Si electrodes. 

The cyclability of pure LTO electrode is shown in Figure 3c–d. For the first discharge and charge cycle at 1 C, capacities of 520 and 266 mAh·g^−1^ were achieved, which resulted in a columbic efficiency of 51%. In the discharge curve, a capacity of about 115 mAh·g^−1^ was delivered as a plateau near 1.5 V and about 390 mAh·g^−1^ was delivered as a long slope between 0.05 and 1.5 V. The plateau at 1.5V represents the phase equilibrium between the two end members Li_4_Ti_5_O_12_ and Li_7_Ti_5_O_12_ [9,37]. In traditional LTO based electrodes test where the cut off voltage is 1 V, most of the capacity is observed in the plateau region [18,19,20,21]. The capacity in the slope region is mainly due to further intercalation of Li_7_Ti_5_O_12_ to Li_8.5_Ti_5_O_12_ and the SEI formation process [38]. During the first charging process, capacity delivered in plateau region at around 1.6 V is around 70 mAh·g^−1^ which is 45 mAh·g^−1^ less than the discharge process. The electrode tends to stabilize at the 20th cycle and the capacity is 118 mAh·g^−1^. The columbic efficiency is near 100% at this point. However, when the charge and discharge curves are compared with the first cycle, it could be seen that the 1.5 V plateaus in both charge and discharge curves diminished and only long slopes were left in the 20th cycle. The capacities decreased when the C-rates increased, however, when the cell was cycled back to 5 C, an increased capacity of 64 mAh·g^−1^ was observed as compared to the previous 5 C test. Unlike pure silicon electrode, the disassembled pure LTO electrode at 220th cycle (in Figure 1e) had a few smaller cracks but the structural integrity was retained, which can be considered as one of the reasons for the total capacity recovery when the cell was charged back at 5 C.

As shown in Figure 3e–f, the first cycle charge and discharge capacities of 35LTO35Si electrode were 955 and 1240 mAh·g^−1^, respectively. In the discharge curve, instead of a plateau, a slope starting from 1.5 to 0.1 V was observed. A long plateau below 0.1 V that had a capacity of 970 mAh·g^−1^ was mainly due to the alloying process of silicon. In the following charging processes, the plateau at 1.6 V still existed, same as pure LTO electrode, and delivered a capacity of 50 mAh·g^−1^. At the 10th cycle under the same C-rate, the discharge capacity decreased to 1004 mAh·g^−1^ and gave a columbic efficiency of 96.6%. The plateau on the charge curve diminished. The capacity achieved at 0.1 C was excellent as compared to both pure silicon and LTO electrodes and proved the positive effect by using these two materials. When C-rates increased from 0.5 to 1 C, the discharge capacity decreased from 581 to 265 mAh·g^−1^, respectively. Although a large amount of the capacity was decreased, 265 mAh·g^−1^ was still comparable to the theoretical capacity of LTO when discharged to 0 V. At an even higher C-rate of 2 C, a capacity of only 10 mAh·g^−1^ was retained. When the cell was charged back to 0.5 C, a recovery of 70% was observed, which is attributed to the stable structure of the composite sample. Another reason, which contributed to the decreased capacity at high C-rates could be its sluggish kinetics from the silicon component. The 50LTO20Si electrode showed similar charge-discharge curves and capacities for the first cycle, as shown in Figure 3g–h. However, unlike 35LTO35Si, when the C-rate increased, even at 1 C, the plateau at 1.6 V did not diminish in the charging curve. At lower C-rates (<1 C), the electrode with lower LTO contents showed higher capacity whereas, at high C-rates (>1 C), higher LTO concentration was helpful to maintain the performance. As for 50LTO20Si at 5 C, 100 mAh·g^−1^ capacity was still achievable. For the disassembled electrode image at 195th cycle in Figure 1f, no obvious cracks were generated in such electrode even after high C-rate testing. The stable capacity at high C-rates could be attributed to the structural integrity of the composite. In Figure 3i–j, the 65LTO5Si electrode shows relatively stable capacities under various C-rates, however, the capacities are not comparable to that of 50LTO20Si and 35LTO35Si because of low concentration of Si. It should be noted that there is no capacity decrease when the cell was charged back at 1 C even after 10 C tests for 20 cycles. In its charge-discharge curves, it should be noted that only slopes instead of plateaus existed in the curves from the very first cycle. Comparing our composite electrodes of 65LTO5Si, 50LTO20Si and 35LTO35Si, the plateaus at 1.5 V in discharge curves and at 1.6 V in charging curves differed from the first cycles and the following cycles, and also varied from low C-rates to high C-rates. However, it is ambiguous (i) if discharging the cell to 0 V had an influence on the 1.5 V plateau region; (ii) if the C-rates had any effect on the plateau and/or (iii) if other active materials affected the plateau. Further studies are required in order to investigate these phenomena.

**Figure 4:**
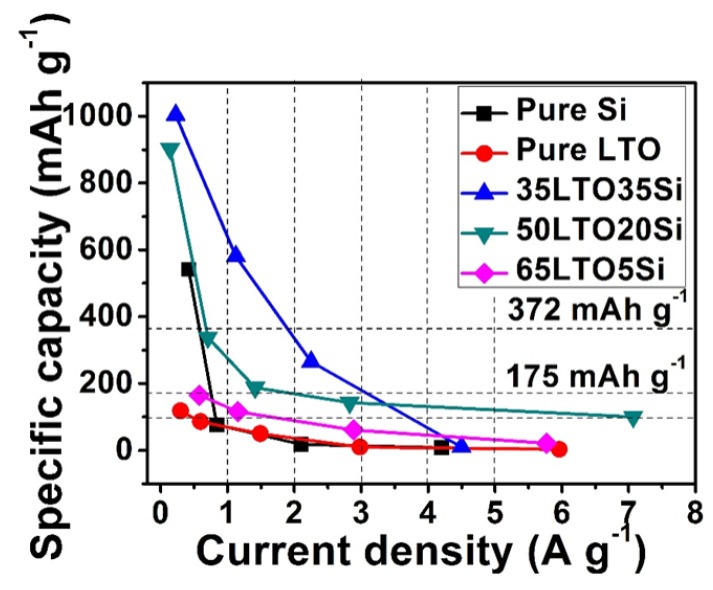
Relationship between capacities and current densities for all electrodes.

Since Si and LTO have a huge difference in theoretical capacities, the current densities of the composite samples at the same C-rate of different samples are greatly varied. As shown in Table 1, the current density of 35LTO35Si at 1 C is about 4 times that of 65LTO5Si for the same C-rate, which makes current density a useful parameter as opposed to the commonly used C-rate to analyze such composite electrodes. Figure 4 shows the relationship between capacity and current density for each sample. The synergism between LTO and silicon is obvious and there is major enhancement in capacities, especially at current densities lower than 3 A·g^−1^. The stability of 65LTO5Si is similar to pure-LTO but the capacity of the electrode is limited due to low Si contents. 50LTO20Si and 35LTO35Si show relatively better overall performance since the capacity enhancement is realized without sacrificing much rate performance. At lower current densities (<3 A·g^−1^), 35LTO35Si performs better than 50LTO20Si due to higher Si involvement. However, when current density is higher than 3 A·g^−1^, 50LTO20Si delivers higher capacity than 35LTO35Si. Even at a high current density of 7 A·g^−1^, a capacity of 100 mAh·g^−1^ is still achievable, which is mainly attributed to the contribution of LTO. Compared to the theoretical capacities of graphite (372 mAh·g^−1^) and LTO (175 mAh·g^−1^ when discharged to 1 V), the performance of both 50LTO20Si and 35LTO35Si were excellent at the current density of 3 A·g^−1^.

From the above results, we have confirmed our strategy to achieve better energy capacity and rate capability by using LTO/Si composites. The addition of Si can enhance the specific capacity of the electrode and at the same time LTO could effectively buffer the volume change of Si, thereby resulting in enhanced C-rate performance. A thin layer of oxide (thickness: 3–5 nm) has been observed in our previous work by TEM using same Si product [32]. The effect of such thin oxide layer on the electrochemical performance could be complicated. At one hand, the oxide layer usually has poor electron conductivity, which could be a limiting factor for the poor rate performance of the Si based battery electrodes [40]. At another hand, it is reported that SiO_2_ and non-stoichiometric silicon oxides are electrochemical active with reasonable Li ion storage capacity [30,41]. How to distinguish the contribution of thin oxide layer is still a big challenge especially in the composite system, which will require more effort in the future work. We believe that great enhancement in the electrochemical performance of the composites can be realized with careful engineering of each component.

## 3. Experimental Section

### 3.1. Sample Preparation and Characterization

Silicon powder (<100 nm, Sigma-Aldrich, St. Louis, MS, USA) and Li_4_Ti_5_O_12_ (D10 = 1.09 μm, MTI corporation, Richmond, VA, USA) powders were used as received, without any further treatment. The composite electrodes were prepared by casting a slurry on copper foil. The slurry was prepared by mixing different amounts of Li_4_Ti_5_O_12_ powder, silicon powder, poly (acrylic acid) (PAA) and Super P Li^®^ in ethanol (See Table 1 for detailed sample composition). PAA and super P Li^®^ were used as binder and conducting additive, respectively. Then the electrodes were transferred to a vacuum oven and dried at 80 °C for 12 h. The morphology of the electrodes was investigated using a field emission-scanning electron microscope (JEOL 6335FE-SEM, Peabody, MA, USA).

### 3.2. Electrochemical Characterization

Electrochemical test cells (2032 coin cells) were assembled in an argon filled glove box (VAC Nexus I, Hawthorne, CA, USA). The composite electrode was used as the working electrode; a lithium foil was used as the counter electrode and reference electrode. Celgard 2400 microporous polypropylene (Charlotte, NC, USA) was used as the separator. The electrolyte was 1 M lithiumbis(perfluoroethylsulfonyl)imide dissolved in ethylene carbonate (EC): diethyl carbonate (DEC): ethyl methyl carbonate (EMC) in the volume ratio of 1:1:1. Cyclic voltammetry (CV) was performed using Bio-logic versatile multichannel potentiostat (VMP3). All electrochemical cells were galvanostatically cycled at room temperature using a NEWARE BTS-610 Battery Test System (Shenzhen, China). All the voltages mentioned in this manuscript are *vs.* Li/Li^+^.

## 4. Conclusions

The novel LTO/Si composite electrodes have been demonstrated as promising anodes for Li-ion batteries. By comparing the electrochemical performance of composite electrodes with different Si and LTO concentrations at diverse current densities, it was shown that the LTO/Si composite electrodes showed both improved energy capacity and rate capability due to the synergy between silicon and LTO. It indicates that the LTO/Si composites benefit from both the high energy capacity of silicon and the good rate capability of LTO. The present finding indicates that it is achievable to improve the specific capacity without sacrificing much rate capability of LTO-based electrodes for Li-ion batteries.
